# Supplementary material for: Hepatic Steatosis and Fibrosis in Chronic Inflammatory Bowel Disease
Source: J Clin Med. 2022 May 6;11(9):2623. doi: 10.3390/jcm11092623 (PMC9105667; doi:10.3390/jcm11092623)
Supplement: Supplementary file 1 [file jcm-11-02623-s001.zip › jcm-1647895-supplementary.pdf]

Journal of
Clinical Medicine

MDPI

**Table S1.** Steatosis.

| | | Variables in the Equation | | | | | | | |
|---|---|---|---|---|---|---|---|---|---|
| | | B | S.E. | Wald | df | Sig. | Exp(B) | 95% C.I.for EXP(B) | |
| | | | | | | | | Lower | Upper |
| Step 1[a] | Age | 0.018 | 0.020 | 0.802 | 1 | 0.371 | 1.018 | 0.979 | 1.059 |
| | BMI | 0.001 | 0.005 | 0.096 | 1 | 0.756 | 1.001 | 0.992 | 1.011 |
| | Duration of disease | 0.072 | 0.027 | 7.247 | 1 | 0.007 | 1.075 | 1.020 | 1.133 |
| | Steroids | 1.211 | 0.764 | 2.513 | 1 | 0.113 | 3.358 | 0.751 | 15.019 |
| | AST | −0.001 | 0.006 | 0.037 | 1 | 0.848 | 0.999 | 0.987 | 1.011 |
| | g_glutamyltransferase | 0.002 | 0.003 | 0.537 | 1 | 0.464 | 1.002 | 0.997 | 1.007 |
| | CRP | −0.008 | 0.006 | 1.452 | 1 | 0.228 | 0.992 | 0.980 | 1.005 |
| | Nicotine use | −0.484 | 0.605 | 0.640 | 1 | 0.424 | 0.616 | 0.188 | 2.017 |
| | Constant | −2.578 | 1.080 | 5.697 | 1 | 0.017 | 0.076 | | |

a. Variable(s) entered on step 1: Age, BMI, Duration of disease, Steroids, AST, gamma_glutamyltransferase, CRP, nicotine us. Multivariate logistic regression method enter; *p* value significance < 0.05; B: Co-efficient for the constant; SE: standard error around the co-efficient for the constant; Wald chi square statistics; df: degree of freedom for Wald chi square statistics; Exp(B): exponentiation of B co-efficient which is an odds ratio (OR); CI:95% Confidence interval for the odds ratio with its upper and lower limits.

**Table S2.** Fibrosis.

| | | Variables in the Equation | | | | | | | |
|---|---|---|---|---|---|---|---|---|---|
| | | B | S.E. | Wald | Df | Sig. | Exp(B) | 95% C.I.for EXP(B) | |
| | | | | | | | | Lower | Upper |
| Step 1[a] | Duration of disease | 0.092 | 0.036 | 6.484 | 1 | 0.011 | 1.096 | 1.021 | 1.176 |
| | AST | 0.001 | 0.018 | 0.003 | 1 | 0.956 | 1.001 | 0.965 | 1.038 |
| | g_glutamyltransferase | 0.001 | 0.004 | 0.067 | 1 | 0.796 | 1.001 | 0.993 | 1.009 |
| | IBD Type | 1.385 | 1.117 | 1.538 | 1 | 0.215 | 3.996 | 0.448 | 35.686 |
| | Hepatomegaly | −0.478 | 0.784 | 0.372 | 1 | 0.542 | 0.620 | 0.133 | 2.880 |
| | ALT | 0.001 | 0.022 | 0.004 | 1 | 0.947 | 1.001 | 0.959 | 1.046 |
| | Constant | −4.998 | 1.379 | 13.135 | 1 | 0.000 | 0.007 | | |

a. Variable(s) entered on step 1: Duration of disease, AST, gamma_glutamyltransferase, IBD Type (Crohn´s disease, ulcerative colitis) Hepatomegaly, ALT. Multivariate logistic regression method enter; *p* value significance < 0.05; B: Co-efficient for the constant; SE:standard error around the co-efficient for the constant; Wald chi square statistics; df:degree of freedom for Wald chi square statistics; Exp(B): exponentiation of B co-efficient which is an odds ratio (OR); CI: 95% Confidence interval for the odds ratio with its upper and lower limits.
